# hTERT promoter activity and CpG methylation in HPV-induced carcinogenesis

**DOI:** 10.1186/1471-2407-10-271

**Published:** 2010-06-09

**Authors:** Jillian de Wilde, Jan M Kooter, Renée M Overmeer, Debbie Claassen-Kramer, Chris JLM Meijer, Peter JF Snijders, Renske DM Steenbergen

**Affiliations:** 1 Department of Pathology, Unit of Molecular Pathology, VU University Medical Center, PO box 7057, 1007 MB Amsterdam, the Netherlands; 2 Department of Genetics, FALW, IMC, Vrije Universiteit Amsterdam, De Boelelaan 1085, 1081 HV Amsterdam, the Netherlands

## Abstract

**Background:**

Activation of telomerase resulting from deregulated hTERT expression is a key event during high-risk human papillomavirus (hrHPV)-induced cervical carcinogenesis. In the present study we examined hTERT promoter activity and its relation to DNA methylation as one of the potential mechanisms underlying deregulated hTERT transcription in hrHPV-transformed cells.

**Methods:**

Using luciferase reporter assays we analyzed hTERT promoter activity in primary keratinocytes, HPV16- and HPV18-immortalized keratinocyte cell lines and cervical cancer cell lines. In the same cells as well as cervical specimens we determined hTERT methylation by bisulfite sequencing analysis of the region spanning -442 to +566 (relative to the ATG) and quantitative methylation specific PCR (qMSP) analysis of two regions flanking the hTERT core promoter.

**Results:**

We found that in most telomerase positive cells increased hTERT core promoter activity coincided with increased hTERT mRNA expression. On the other hand basal hTERT promoter activity was also detected in telomerase negative cells with no or strongly reduced hTERT mRNA expression levels. In both telomerase positive and negative cells regulatory sequences flanking both ends of the core promoter markedly repressed exogenous promoter activity.

By extensive bisulfite sequencing a strong increase in CpG methylation was detected in hTERT positive cells compared to cells with no or strongly reduced hTERT expression. Subsequent qMSP analysis of a larger set of cervical tissue specimens revealed methylation of both regions analyzed in 100% of cervical carcinomas and 38% of the high-grade precursor lesions, compared to 9% of low grade precursor lesions and 5% of normal controls.

**Conclusions:**

Methylation of transcriptionally repressive sequences in the hTERT promoter and proximal exonic sequences is correlated to deregulated hTERT transcription in HPV-immortalized cells and cervical cancer cells. The detection of DNA methylation at these repressive regions may provide an attractive biomarker for early detection of cervical cancer.

## Background

Telomerase is a ribonucleoprotein with reverse transcriptase activity that adds hexameric TTAGGG repeats onto the telomeric ends of chromosomes. Thereby it compensates for telomere shortening that is inherent to DNA replication, the so-called end replication problem [1]. Telomerase activation is a key feature of human cancers. The telomerase complex consists of several subunits, including a structural RNA component (hTR) that serves as a template during telomere elongation [2] and a catalytic subunit (hTERT) [3]. hTR is ubiquitously expressed and frequently elevated in cancer cells [4]. Expression of hTERT is restricted to telomerase positive cells [3], indicating that hTERT expression controls telomerase activity. Indeed, ectopic expression of hTERT is sufficient for the activation of telomerase in telomerase negative cells [5].

The hTERT gene has a CG-rich, TATA-less promoter, within which a proximal region of 200 base pairs has been identified as the core promoter, being indispensable for transcription activation [6,7]. This core sequence contains numerous binding sites for both transcriptional activators and repressors [6,8-10].

The fact that the 5' region of the hTERT gene and its promoter are part of a large ~4 kb CpG island (-1800 to +2200, numbered relative to the ATG) implies that hTERT expression may be responsive to CpG methylation [8]. To evaluate this possible regulatory mechanism, several groups have assessed the methylation status of the hTERT CpG island in a variety of primary cells as well as immortal and cancer cell lines [11-17]. In embryonic stem cells and normal lymphocytes, which express hTERT, almost all CpGs around the transcription start site of the hTERT gene are unmethylated [17]. However, hTERT expression is not always associated with hypomethylation. In many cancer cell lines hTERT expression is often correlated with hypermethylation [11,14], although the expression level in these cells is usually much lower than in ES cells [17]. Other studies did not find a clear correlation between hTERT methylation and hTERT expression [12,13]. Zinn *et al. *[17] showed that in most cancer cell lines the hTERT promoter is densely methylated, but that CpGs around the transcription start site of a substantial number of hTERT alleles is unmethylated. This suggests that methylation of hTERT promoters is heterogeneous and that only unmethylated alleles are expressed. Indeed, histones at a hypomethylated core-promoter carry transcription active marks (H3K9Ac and H3K4 me2) whereas a methylated core-promoter carries inactive marks (H3K9 me3 and H3K27 me3) [17]. The importance of this core promoter region is also illustrated by the observation that sequences surrounding the hypomethylated and active core promoter may be hypermethylated [18]. In addition to the importance of the methylation status of the core-promoter region, other sequences in hTERT CpG island may also be important. Choi *et al. *[11] observed in colorectal cancer hypermethylation of a specific CpG outside known transcription factor binding sites, that together with hypermethylation of a number of CpGs closer to the transcription start site was associated with increased hTERT expression. It is unknown how methylation of these CpGs induce hTERT expression. The CpG rich region encompassing the 5' proximal region of the gene has also been shown to affect hTERT expression [18]. This region binds the transcriptional repressor CTCF and in cancer cells CTCF repression was shown to be relieved by methylation of its binding site. A second CTCF binding site has been identified in exon 2 [18]. Thus, aberrant methylation of the 5' end of gene, including CTCF sites, can affect hTERT expression.

Despite the fact that deregulated hTERT expression is implicated in human papillomavirus (HPV)-mediated pathogenesis of cervical cancer, little is known about the mechanism and whether it also involves changes in CpG methylation.

Infection with high-risk human papillomavirus (hrHPV) has been recognized as the main risk factor for the development of cervical cancer, which is supported by the detection of hrHPV in virtually all cervical carcinomas [19,20]. HrHPV activities together with host cell factors are required for the progression of hrHPV-induced precancerous lesions to invasive cancer.

Our previous studies showed elevated hTERT mRNA levels and increased telomerase activity in over 90% of cervical carcinomas and less than half of high-grade precursor lesions, so-called cervical intraepithelial neoplasias (CINs). Most normal cervical tissues and low-grade CIN lesions were devoid of detectable hTERT mRNA and telomerase activity [21]. These findings indicate that elevated levels of hTERT mRNA reflect a late step in the CIN to carcinoma sequence following hrHPV infection. It is conceivable that telomerase-positive CIN lesions have gained an immortal phenotype and have reached a point of no return in terms of malignant potential. Ectopic expression of HPV16 E6, along with E6AP can, (in part) mediated by c-Myc, induce hTERT transcription and telomerase activity directly [22-24].

Additionally, E6 associated with E6AP can degrade the hTERT repressor NFX1-91, which normally interacts with the co-repressor complex mSin3A/histone deacetylase (HDAC) at the hTERT promoter [25]. By degrading NFX1-91, E6/E6AP changes the chromatin structure at the hTERT promoter which results in increased hTERT expression [26]. More recently, NFX1-123, which like NFX1-91 is a splice variant of NFX1, has been demonstrated to increase hTERT expression posttranscriptionally in HPV E6 containing keratinocytes [27].

However, several other studies have shown that in the context of the whole HPV genome, E6 activity alone is insufficient for increasing hTERT expression and concomitant telomerase activation in human keratinocytes [28,29]. Microcell-mediated chromosome transfer studies have shown that the increased hTERT mRNA expression and telomerase activity in HPV-immortalized cells and cervical cancer cells may depend on a recessive event, suggesting inactivating alterations of host cell genes being involved in this process as well [29-31]. We previously demonstrated that potential hTERT regulatory genes reside on chromosome 6q, which may either directly or indirectly regulate hTERT transcription [5,32].

In the present study we aimed to directly assess the activity of the regulatory hTERT sequences in HPV-transformed cells and the potential role of hTERT methylation in cervical carcinogenesis. To this end, we first analyzed the activity of various hTERT promoter and proximal exonic/intronic sequences by transient luciferase reporter assays in four human keratinocyte cell lines, immortalized by full-length HPV16 and HPV18 DNA, and three cervical cancer cell lines. Secondly, we used extensive bisulfite sequencing and quantitative methylation-specific PCR (qMSP) to examine hTERT DNA methylation in all cell lines as well as cervical tissue specimens representing the complete spectrum of (pre-)malignant disease.

## Methods

### Cells and cell lines

Primary keratinocytes (EK94-2 and EK04-3) were isolated from human foreskins, as described before [29]. The cell lines FK16A, FK16B, and FK18A and FK18B were established by transfection of primary foreskin keratinocytes with the entire HPV16 and HPV18 genomes, respectively [29]. The cervical carcinoma cell lines SiHa (HPV16), HeLa (HPV18) and CaSki (HPV16), were obtained from the American Type Culture Collection (Manassas, VA). Culture conditions for these cells and cell lines were described previously [29,33].

The hTERT-negative osteosarcoma cell line Saos-2 was obtained from American Type Culture Collection (Manassas, VA) and cultured in Dulbecco's modified Eagle medium (DMEM) supplemented with 10% foetal bovine serum (FBS), penicillin (100 U/mL), streptomycin (100 μg/mL), and L-glutamine (2 mM) (all from Life Technologies, Inc., Breda, The Netherlands).

### Clinical samples

Formalin fixed, paraffin-embedded biopsies of normal cervix (n = 22), CIN1 lesions (n = 26), CIN3 lesions (n = 29) and cervical squamous cell carcinomas (SCCs) (n = 21) were collected during routine clinical practice and stored at the Department of Pathology of the VU University Medical Center. Cervical scrapings analyzed by bisulfite sequencing analysis were collected from women taking part in a population-based cervical screening trial [34]. We included three scrapings of women without CIN disease (classified as normal cytology n = 2, and borderline mild dyskaryosis n = 1) and one scrape (classified as moderate dyskaryosis) of a woman with CIN disease.

HPV DNA presence was determined using GP5+/6+-PCR, followed by an enzyme immunoassay (EIA) with cocktail probes representing pools of high- and low-risk HPV types, respectively [35]. Subsequently, HPV typing was performed on PCR products of EIA-positive cases using reverse line blot (RLB) analysis, as described previously [36]. EIA-positive samples that failed to reveal an RLB signal, were considered to contain HPV (sub)types or variants that do not react with the specific RLB probes and these HPVs were referred to as HPV X (X). All cervical scrapings, 7 CIN 1 lesions, all except one CIN3 lesions, and all SCCs were hrHPV-positive by GP5+/6+-PCR. None of the normal biopsies was hrHPV-positive. This study was approved by the Institutional Review Board of the VU University Medical Center. Cervical scrapings were obtained from the population-based cervical screening trial POBASCAM, registered as an International Standard Randomized Controlled Trial under number ISRCTN20781131 [34].

### RNA isolation and quantitative RT-PCR

Total RNA was isolated from cell lines using RNA Bee (Tel-test, Friendswood, TX) following the manufacturer's instructions. hTERT mRNA was quantified by real time RT-PCR using the TeloTAGGG hTERT kit (Roche, Woerden, The Netherlands), according to the manufacturer's manual. Relative hTERT levels were determined by dividing hTERT expression levels by expression levels of the housekeeping gene Porphobilinogen Deaminase (PBGD), which was also included in the TeloTAGGG hTERT kit (Roche). hTERT levels were considered to be low when the ratio between hTERT and PBGD expression was < 1. All PCR reactions were performed in duplicate and mean values including the standard deviation were calculated.

### hTERT promoter constructs

The pGL3-control vector (Promega Benelux BV, Leiden, The Netherlands) contains the Firefly luciferase gene under the control of a CMV promoter. The pGL3-basic vector (Promega Benelux BV) is a promoterless vector containing the Firefly luciferase gene. The hTERT reporter constructs contain DNA fragments of the hTERT promoter inserted upstream of the Firefly luciferase gene in the pGL3 basic vector.

hTERT-1821/-23, hTERT-499/-23, hTERT-297/-23, hTERT-297/+81, and hTERT-297/+357, which contain hTERT promoter sequences from -1821 bp to -23 bp, -499 to -23 bp, -297 to -23 bp, -297 to +81 bp, and -297 to +357 bp, respectively, relative to the ATG start site, have previously been referred to as pTERT-1821, pTERT-499, pTERT-297, pTERT-297/ex1 mini, and pTERT-297/ex1 [18,37] and were kindly provided by Dr. J. Benhattar (Institute of Pathology, Centre Hospitalier Universitaire Vaudois, Lausanne, Switzerland).

### Transfection and luciferase assay

Cells were seeded on 48 wells plates at a concentration of 10,000 cells/well (SiHa, HeLa and CaSki) or 20.000 cells/well (EK04-3, FK16A, FK16B, FK18A, and FK18B) and cultured overnight. Transient transfection of luciferase reporter plasmids was performed using TransPEI (Eurogentec, Seraing, Belgium) according to the manufacturer's protocol. The Renilla luciferase reporter vector (Promega Benelux BV) was co-transfected as an internal control for transfection efficiency. Briefly, cells were exposed to a transfection mixture containing 500 ng of reporter plasmid and 25 ng of internal control for 4 hours at 37°C.

Cells were harvested 24 hours after transfection. Luciferase assays were performed using the Dual Luciferase Reporter Assay System (Promega Benelux BV) and a Lumat LB 9507 luminometer (EG&G Berthold, Bad Wildbad, Germany). For calculations the mean values of relative luciferase activity, i.e. Firefly luciferase divided by Renilla luciferase activity, were used, in which the mean relative luciferase activity of the pGL3 control vector was set at 100%. All experiments were performed (at least) in duplicate and mean values including standard deviation were calculated.

### DNA isolation and bisulfite modification

Genomic DNA from cultured cells was isolated using DNA STAT-60 (Tel-Test, Friendswood, TX). Genomic DNA from paraffin-embedded tissue specimens was isolated as described previously [38]. Ten paraffin-embedded tissue sections of 5 μm were digested in 450 μL proteinase K buffer [100 mmol/L Tris-HCl (pH 9.0), 10 mmol/L NaCl, 1% SDS, and 5 mmol/L EDTA] with 50 μL Proteinase K (1 mg/mL; Boehringer-Ingelheim, Alkmaar, The Netherlands) for 24 hours at 52°C with shaking. Thereafter, DNA was isolated by phenol/chloroform/isoamylalcohol (25:24:1) extraction, followed by ethanol precipitation. DNA was dissolved in H_2_O. The histology of sections used for DNA isolation was checked according to the sandwich method in which the first and last section were stained by H&E and checked by a pathologist for presence of the lesion. Tumor sections contained in general 50-80% of tumor cells, whereas sections of CIN lesions contained 10-50% of dysplastic cells.

Genomic DNA of cervical scrapings was isolated using the high pure PCR template preparation kit (HPPTP, Roche, Mannheim, Germany). After a classic cervical smear was made on a slide, cervical scrapes were collected by placing the brush in 5 ml sterile phosphate-buffered saline (PBS, 0.82% (w/v); NaCl, 0.19% (w/v); Na_2_HPO_4_·2H_2_O,·0.03% (w/v); NaH_2_PO_4_·2H_2_O, adjusted to pH 7.4 with HCl) 0.005% merthiolate. Upon arrival in the laboratory, cells were pelleted at 300 *g *for 10 min and resuspended in 1 ml 10 mM Tris-HCl (pH 7.4). A 100-μl Tris-HCl suspension was used for DNA isolation by the HPPTP kit (Roche) according to the recommendations of the manufacturer, except that samples were eluted with 100 μl of elution buffer. Ultimately, 10 μl of eluate was used for HPV detection and 500 ng of DNA for bisulfite modification.

Sodium bisulfite modification, which induces chemical conversion of unmethylated cytosines into uracils, whereas methylated cytosines are protected from this conversion, was performed using the EZ DNA Methylation Kit, according to the manufacturer's guidelines (Zymo Research, Orange, CA).

### Bisulfite sequencing

The hTERT promoter and first exon were amplified using 4 primer sets spanning the hTERT promoter and coding sequence from -442 to +566 bp, relative to the ATG start codon (Table 1). All primers have been previously described by Guilleret *et al. *[39], but were in some cases used in a different combination.

**Table 1:** Primers and probes used for bisulfite sequencing and MSP analysis

Sequence primers/ Location	Sequences
hTERT S1/-442 to -219	F 5' GGGTTATTTTATAGTTTAGGT 3'
	R 5' AATCCCCAATCCCTC 3'
hTERT S2/-208 to +104	F 5' GTTTTGTTTTTTTATTTTTTAGTTT 3'
	R 5' CCAACCCTAAAACCCCAAA 3'
hTERT S3/+88 to +348	F 5' TTTGGGGTTTTAGGGTTGG-3'
	R 5' AACCACCAACTCCTTCAAA-3'
hTERT S4/+319 to +566	F 5' GTAGGTGTTTTGTTTGAAGGA-3'
	R 5' TACCAACAAATAAACCAAC-3'

**MSP primers/** **Location**	Sequences*

hTERT M1/-317 to -262	F 5' TAGATTTT**C**GGGTT**C**GTT**C**G 3'
	R 5' TCTATACCC**G**C**G**AATCCACT 3'
	P 5' C**G**ACCTAACCCC**G**ACAAC**G**CAACTA 3'
hTERT M2/+288 to +419	F 5' GAGTAG**C**GTAGG**C**GATTTAGGG**C**GT 3'
	R 5' **G**TCCAACAAC**G**C**G**AAACC**G**AA 3'
	P 5' C**G**CACAACCTCTACAACACTC**G**AACCACCAACTC 3'
β actin	F 5' TGGTGATGGAGGAGGTTTAGTAAGT 3'
	R 5' AACCAATAAAACCTACTCCTCCCTTAA 3'
	P 5' ACCACCACCCAACACACAATAACAAACACA 3'

*Methylation specific CpGs are printed in bold

The PCR mixtures contained 50 ng of modified DNA, 0.5 μM primers, 200 μM dNTPs, 1.5 mM MgCl_2 _and 1 U of FastStart Taq DNA polymerase (Roche). Amplification conditions were: denaturation at 95°C for 4 minutes; 40 cycles of 95°C for 1 minute, 60°C for 1 minute and 72°C for 1 minute; and a final elongation step of 72°C for 4 minutes. Subsequently, PCR products were purified using a QIAgen PCR purification kit (Westburg, Leusden, The Netherlands) and cloned in pGEM-T using the pGEM-T Easy Cloning kit (Promega). For most regions, between 10 and 20 cloned PCR fragments were sequenced using the BigDye Terminator v1.1 Cycle Sequencing Kit on an ABI Prism Avant Genetic Analyzer (Applied Biosystems, Nieuwerkerk a/d IJssel, The Netherlands) and sequences were analyzed using Vector NTI (Invitrogen, Breda, The Netherlands).

### Quantitative methylation-specific PCR (qMSP)

qMSP was performed using two primer and Taqman probe combinations, one of which was located in the promoter (region 1) and the other in the first intron and exon 2 (region 2) (Table 1). As indicated in bold in Table 1 all primer and probes contained two to four CpG dinucleotides, to ensure specific detection of methylated DNA. A standard curve of bisulfite-treated DNA of SiHa was included in each qMSP. As negative controls, H_2_O, unmodified genomic DNA obtained from SiHa cells and unmethylated DNA obtained from primary keratinocytes were included. The house keeping gene β-actin was included as a quality control (Table 1) [40,41]. qMSP reactions were carried out in a 12 μl reaction volume containing 50 ng of bisulfite-treated DNA, 417 nM of each primer, 208 nM probe and 1× QuantiTect Probe PCR Kit master mix (Qiagen, Westburg, Leusden, The Netherlands) using the ABI 7500 Fast Real-Time PCR System (Applied Biosystems, Nieuwerkerk a/d IJssel, The Netherlands). For each qMSP assay the threshold was fixed at 0.01. All samples analysed in the study showed an ACTB CT value that was equal to or below 31 (CT ≤ 31). qMSP values of target genes were adjusted for DNA input by expressing the results as ratios between the absolute target measurement and the ACTB measurement (mean quantity of methylated hTERT DNA/mean DNA quantity for β-actin * 1000).

All samples were tested in duplicate and in case of discrepancy (in < 10% of cases) in quadruplicate. Samples scored positive if at least two test results (quantity ratios) were above zero.

## Results

### hTERT mRNA expression and hTERT promoter activity are partially correlated

**Figure 1:**
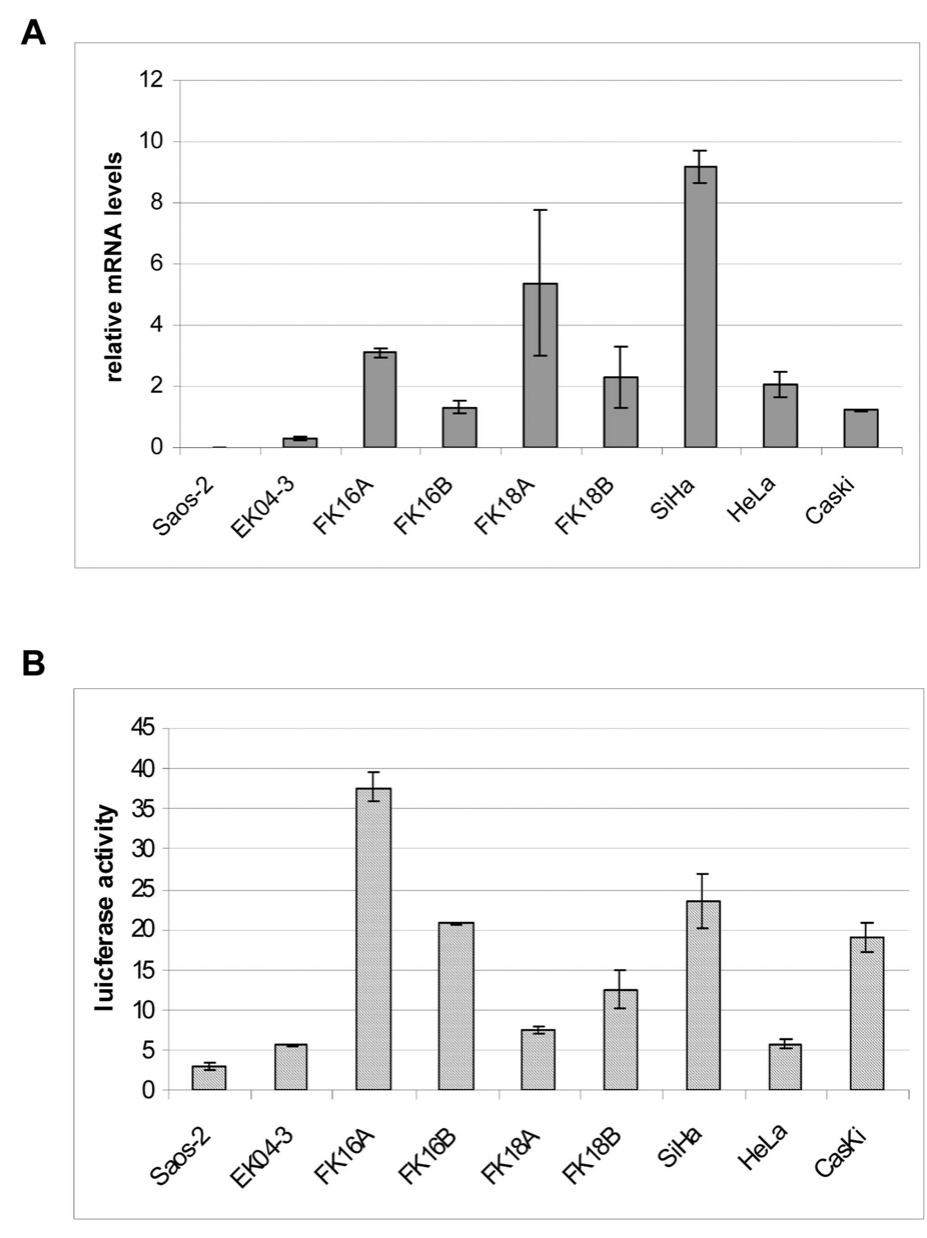
**hTERT mRNA expression and core promoter activity in all cell cultures**. (A) hTERT mRNA levels relative to the housekeeping gene PBGD and (B) hTERT core promoter activity as assessed by the hTERT-297/-23 reporter construct. Given values are percentages of luciferase activity relative to pGL3 control that was set at 100%.

We determined hTERT mRNA expression by real time RT-PCR in primary keratinocytes (EK cells), HPV-immortalized cell lines and cervical carcinoma cell lines SiHa, HeLa, and CaSki. The cell line Saos-2 was included as an hTERT negative control. The HPV-immortalized cell lines comprised two HPV16-transfected keratinocyte cell lines (FK16A and FK16B) and two HPV18-transfected keratinocyte cell lines (FK18A and FK18B). Eventually cell lines became immortal and gained strong telomerase activity, but were not yet tumorigenic in nude mice [29,33]. As shown in Figure 1A, hTERT mRNA was undetectable in Saos-2 cells and present at very low levels in primary keratinocytes (0.2). Relatively high hTERT mRNA levels were detected in all HPV-immortalized cell lines and cervical carcinoma cell lines, with hTERT mRNA levels ranging from 1.2 to 9.1, relative to the housekeeping gene PBGD.

To investigate hTERT promoter activity in these cells, we measured luciferase expression of construct hTERT-297/-23, containing hTERT promoter sequences from -297 to -23, relative to the ATG. hTERT promoter activities were related to the CMV-promoter activity in the pGL3 control vector, which was set at 100%. The relative hTERT promoter activity was 3% in Saos-2 cells and 5% in EK cells (Figure 1B). Relative levels of hTERT promoter activity in *in vitro *immortalized cells and cancer cells ranged from 5% in HeLa cells to 37% in FK16A cells (Figure 1B). However, relative levels of promoter activity did not always correlate with relative hTERT mRNA levels. Promoter activity in EK and Saos-2 cells was comparable to that in HeLa cells (Figure 1B), despite the fact that hTERT mRNA levels were markedly higher in HeLa cells (Figure 1A).

### Repression of hTERT promoter activity by sequences flanking the hTERT core promoter

**Figure 2:**
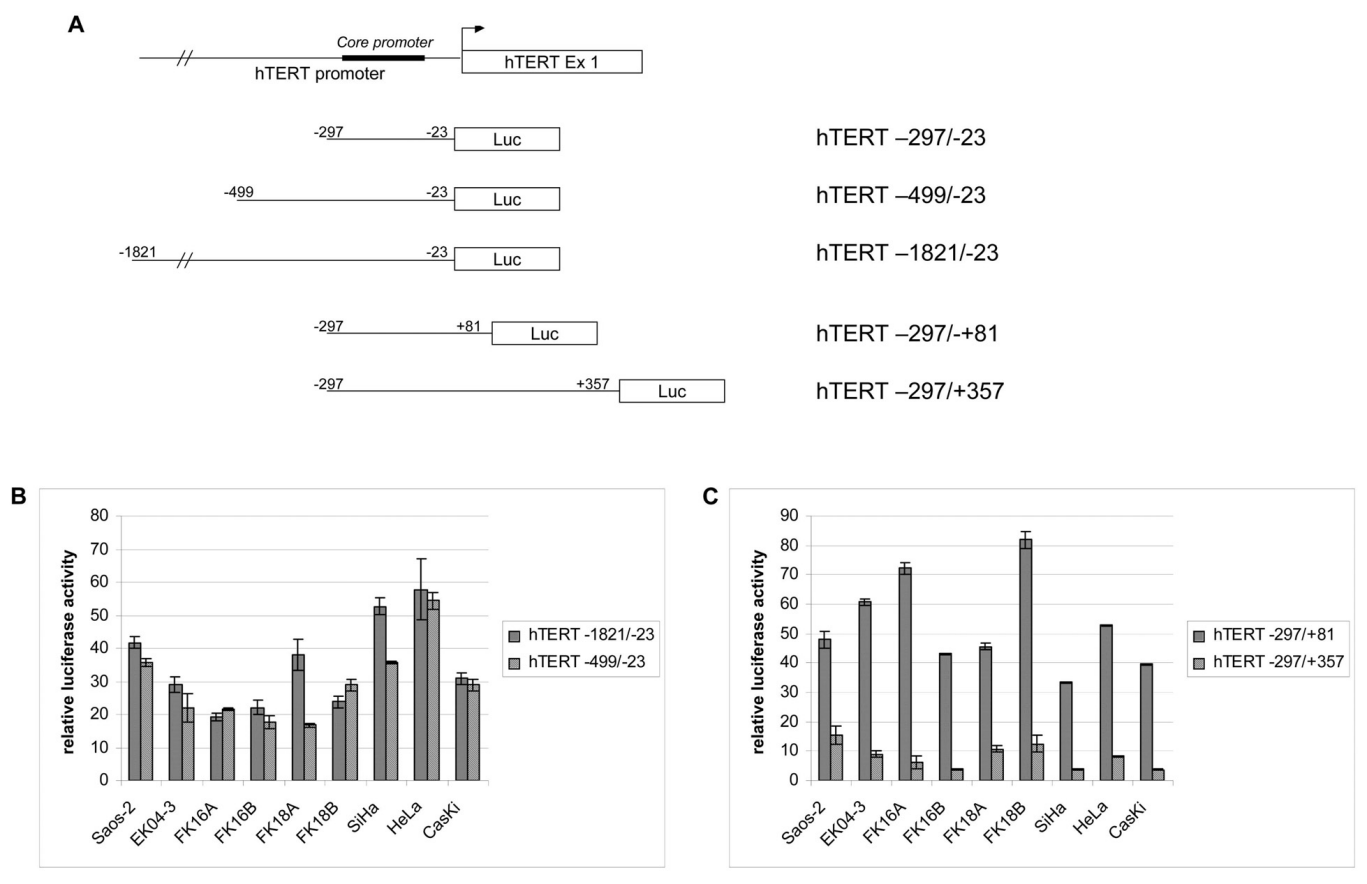
**hTERT promoter activity analysis, using hTERT reporter constructs carrying different promoter and proximal exonic sequences**. (A) Schematic representation of different reporter constructs used. (B) Luciferase activity in Saos-2, EK04-3, FK16A, FK16B, FK18A, FK18B, SiHa, HeLa and CaSki cells following transfection of hTERT-1821/-23 and hTERT-499/-23, relative to hTERT-297/-23 that was set at 100%. (C) Luciferase activity in Saos-2, EK04-3, FK16A, FK16B, FK18A, FK18B, SiHa, HeLa and CaSki following transfection of hTERT-297/+81 and hTERT-297/+357, relative to hTERT-297/-23 that was set at 100%.

To examine the contribution of sequences located 5' and 3' of the hTERT core promoter to hTERT promoter activity, we tested the reporter constructs hhhhTERT-1821/-23, hTERT-499/-23, hTERT-297/+81, and hTERT-297/+357 (schematically represented in Figure 2A) next to the core promoter construct in the various cell lines.

Insertion of 5' sequences, i.e. -499 to -23 (hTERT -499/-23) resulted in a strong reduction in luciferase activity, varying from 45% in HeLa cells to 82% in FK16B and FK18A cells. Also in primary keratinocytes and telomerase negative Saos-2 cells, luciferase activity was strongly repressed, indicating that the repression is cell type-independent. Insertion of additional upstream promoter sequences starting at -1821 (hTERT-1821/-23) did generally not further suppress the promoter activity, indicating that the suppressive effect can mainly be attributed to sequence -499 to -297, relative to ATG. The repression of core promoter activity upon inclusion of both 5'sequences is shown in Figure 2B, in which the luciferase activity of both constructs relative to the hTERT-297/-23 core promoter construct is depicted.

Inclusion of sequences downstream of the core promoter encompassing part of the first exon up to nucleotide +81 (hTERT-297/+81), also repressed the basal luciferase activity, which varied from an 18% reduction in FK18B cells to a 67% decrease in SiHa cells (Figure 2C). A further, very strong repression of promoter activity was seen upon addition of sequences up to +357, spanning the first exon, first intron and part of exon 2 (hTERT-297/+357). The resulting reduction in promoter activity ranged from 85% in Saos-2 cells to 96% in FK16B, SiHa and CaSki cells. The repression of core promoter activity upon inclusion of both 3'sequences is shown in Figure 2C by plotting luciferase activity of both constructs relative to the hTERT-297/-23 core promoter construct.

Conclusively, our findings indicate that both the -499 to -297 region and core promoter downstream exonic/intronic sequences up to +357 contain putative repressive regulatory sequences.

### hTERT bisulfite sequencing analysis in cultured cells

**Figure 3:**
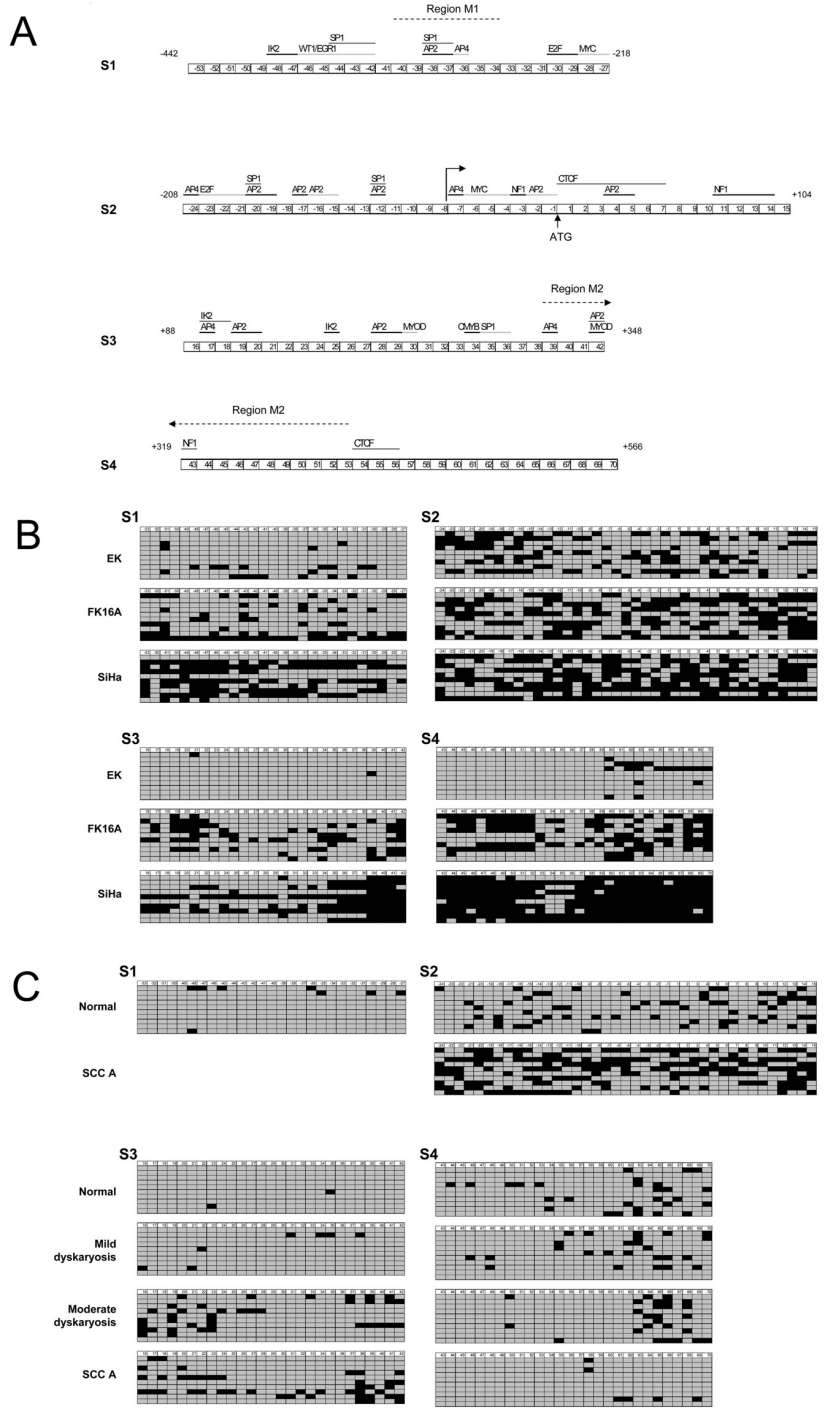
**Bisulfite sequencing results of hTERT regulatory regions**. (A) Schematic overview of the four regions analyzed: S1: -442 to -219, S2:-208 to +104, S3: +88 to +348, and S4:+319 to +566. Numbers refer to the respective CpG dinucleotides and their position relative to the ATG. The positions of transcription factor binding sites, if containing CpG dinucleotides, are shown at the top by the black lines (or grey lines when located directly next to each other). Regions analyzed by qMSP (M1 and M2) are indicated by dotted lines. (B) CpG methylation results of individual cloned PCR products of cultured cells (EK cells, FK16A cells, and SiHa cells). (C) CpG methylation results of individual cloned PCR products of clinical specimens (cervical scraping classified as normal (Pap1), borderline mild dyskaryosis (BMD, Pap2/3a1), moderate dyskaryosis (Pap3a2), and a cervical squamous cell carcinoma (SCC)). Of all samples ten representative clones are shown. Unmethylated CpGs are indicated by grey squares and methylated CpGs are indicated by black squares.

Since in telomerase positive cells the suppressive effect of sequences flanking the core promoter seems to be released, we reasoned that this might be due to epigenetic differences. This view is supported by studies that show a correlation between hTERT promoter methylation and hTERT transcription [11,14,39]. We therefore determined the methylation status of the putative hTERT regulatory sequences identified in the transient expression assays. By bisulfite sequencing we analyzed CpG methylation of the region spanning nucleotides -442 to +566, relative to the ATG in primary keratinocytes (EK), FK16A and SiHa cells. Using overlapping PCR products the following 4 regions were analyzed: -442 to -219 bp (region S1), -208 to +104 bp (region S2), +88 to +348 bp (region S3), and +319 to +566 bp (region S4), all relative to the ATG site (Figure 3A).

Throughout the promoter, except for region S2, few methylated CpGs were detected in EK cells. Compared to these primary cells, methylation was increased in the HPV16-immortalized cell line FK16A at all four regions. An even higher methylation level was found in S1, S3 and S4 from SiHa cells. An overview of the distribution and frequency of methylated CpGs in individual alleles in EK, FK16A and SiHa is shown in Figure 3B.

To determine whether methylation occurs at specific transcription factor binding sites, the known CpG overlapping binding sites were plotted at the top of Figure 3A. A rather random distribution of methylated CpGs relative to these sites was evident within region S1 and S2 (Figure 3B, top panels). Interestingly, the presence of a so-called unmethylated core, including the CpGs -19 to -12, which has been described to contribute to hTERT expression by others [17,42], was not observed in the hTERT expressing cells. Within region S3 methylation of FK16A and SiHa cells was mainly concentrated at the 3' end. The 3' sub-region preferentially affected by methylation in these cell lines, contains putative binding sites for the transcription factors AP2, AP4 and MYOD [10]) (Figure 3B, lower left panel). Methylation at region S4 was most extensive in SiHa cells, followed by FK16A cells (Figure 3B, lower right panel). Interestingly, within this region the lowest methylation was seen at the second CTCF binding site (CpG 54-56) in all cells analyzed. However, at the first CTCF binding site located within region S2, methylation was slightly increased (Figure 3B, lower right panel).

Next to these three cell cultures, the remaining three HPV-immortalized cell lines (FK16B, FK18A and FK18B) as well as the cervical cancer cell lines HeLa and CaSki were subjected to bisulfite sequencing analysis of the S3 and S4 region. Similar to FK16A and SiHa cells, it was found that all remaining three HPV-immortalized cell lines and the two cervical cancer cell lines showed a high density of methylated CpGs in these two regions compared to primary keratinocytes. Moreover, in SiHa, FK16A and FK16B cells methylation at the 3'end of the S3 region was particularly high.

A summary of CpG methylation frequencies in regions S1 to S4 is shown in Table 2.

**Table 2:** Percentage of methylation in cultured primary keratinocytes, HPV-immortalized cell lines and cervical carcinoma cell lines

Cell culture	%methylation (total number of clones analyzed)			
	S1 (-442/-219)	S2 (-208/+104)	S3 (+88/+348)	S4 (+319/+566)
EK	9 (20)	29 (20)	0 (15)	6 (22)
FK16A	27(20)	53 (20)	26 (13)	47 (18)
SiHa	51(20)	53 (20)	45 (15)	83 (22)

FK16B			27 (16)	40 (16)
FK18A			40 (16)	38 (24)
FK18B			21 (13)	41 (20)
HeLa			18 (12)	29 (21)
CaSki			65 (6)	93 (20)

### hTERT bisulfite sequencing analysis in clinical specimens

In addition to the cell lines, DNA from four hrHPV-positive cervical scrapings, classified as normal cytology (n = 2), borderline or mild dyskaryosis (n = 1) and moderate dyskaryosis (n = 1), respectively, as well as a cervical carcinoma biopsy were analyzed by bisulfite sequencing at regions S3 and S4. An overview of methylation of individual CpGs and locations of transcription factor binding sites is shown in Figure 3C. Methylation frequencies in region S3 and S4 are summarized in Table 3.

**Table 3:** Percentage of methylation in hrHPV-positive cervical scrapings and a cervical carcinoma

Cervical specimen	% methylation (number of clones analyzed)	
	S3	S4
Normal cytology, hrHPV+	1 (23)	10 (24)
Normal cytology, hrHPV+	1(25)	11(25)
Borderline mild dyskaryosis, hrHPV+	3 (20)	8 (20
Moderate dyskaryosis, hrHPV+	13 (20)	8 (20)
Cervical carcinoma, HPV16+	17 (20)	7 (20)

Methylation levels in clinical specimens were generally lower than in cell cultures, most likely resulting from the admixture of normal cells in these samples. Only at S3 an increase in methylation was detected proportional to increased severity of the cellular abnormality (Figure 3C, lower left panel). If present, methylation within region S3 tended to be highest at both the 5'and 3' ends, which together cover putative AP2, AP4 and MYOD binding sites, as mentioned above [10]. Although methylation of the S4 region was not increased in the cancer specimen compared to the normal specimens, it was, when present, concentrated at the 3' end of the region (Figure 3C, lower right panel).

Thus, increased methylation of particularly the S3 region, spanning +88 to +348, was associated with malignant transformation both *in vitro *and *in vivo*. This particular region resides in the fragment that displayed the strongest repressive effect on the core promoter activity in the transient expression assays.

### qMSP analysis of the hTERT promoter and first exon

To further assess the hTERT methylation status in cervical tissue specimens, we designed two quantitative methylation-specific PCRs (qMSPs). With primer design restrictions taken into account, these qMSPs encompassed sequences that showed increased methylation in immortalized cell lines and/or severe cervical disease, as found by bisulfite sequencing; i.e. one within the proximal promoter region (M1: -317 to -262) and a second within the gene (M2: +288 to +419) (Figure 3A).

**Figure 4:**
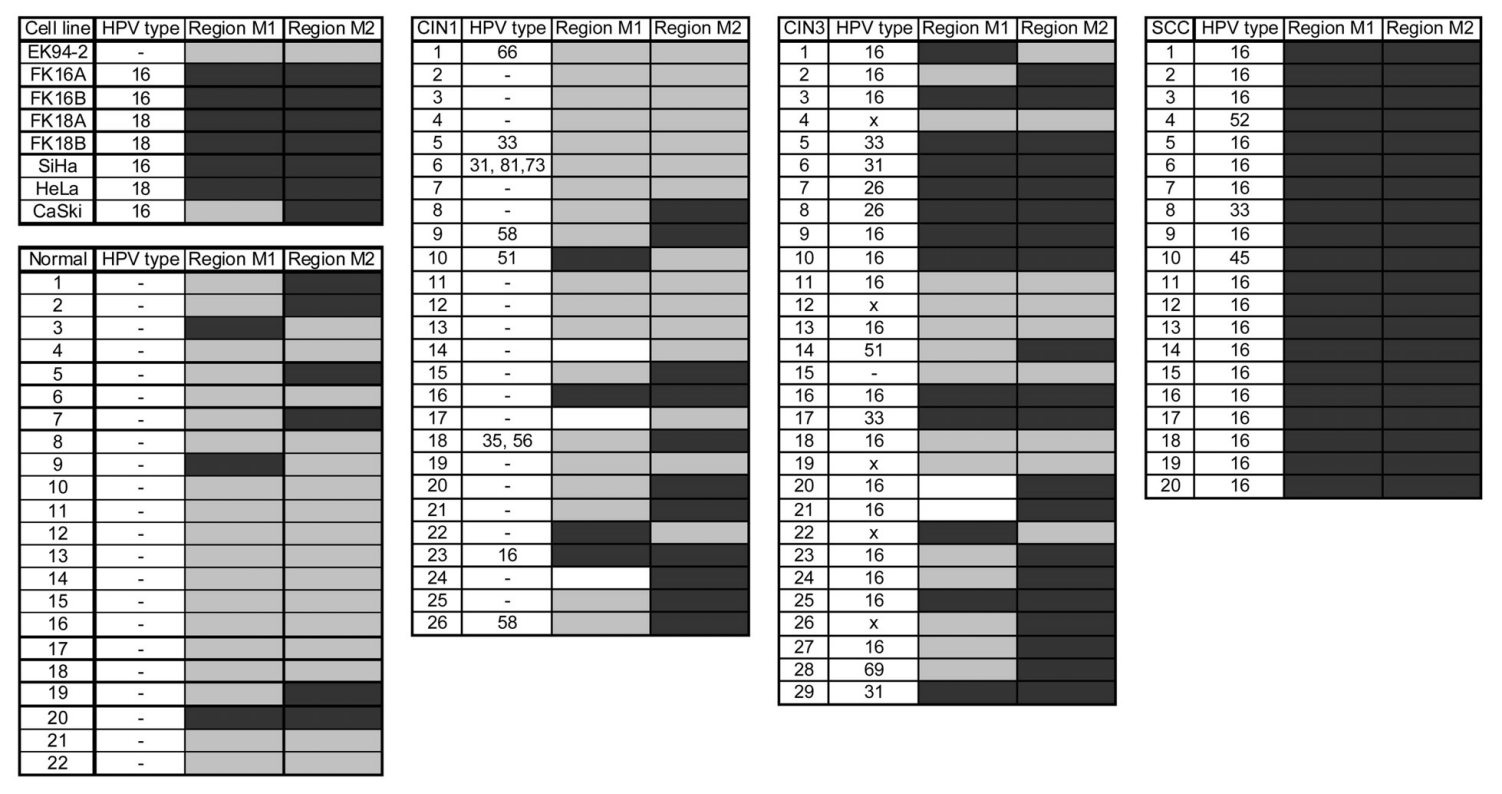
**Quantitative methylation-specific PCR (qMSP) results for two regions of the hTERT gene**. Results of both cell cultures and cervical tissue biopsies are shown. Region M1 spans nts -317 to -262 bp, relative to the ATG and region M2 spans nt +288 to +219 bp, relative to the ATG. Black boxes indicate methylation positive, gray boxes indicate no methylation and white boxes remain undetermined. HPV status of specimens is depicted as specific HPV type present or negative (-).

For validation we tested primary keratinocytes (EK94-2), having a very low hTERT mRNA expression and little methylation as assessed by bisulfite sequencing, and HPV-immortalized and cervical cancer cell lines with elevated hTERT mRNA levels and methylation of the hTERT CpG island, i.e. FK16A, FK16B, FK18A, FK18B, SiHa, HeLa and CaSki. In line with the bisulfite sequencing results, primary keratinocytes were negative by qMSP at both regions, while all hTERT-positive cell lines, except CaSki, were qMSP-positive at both regions. CaSki tested negative at region M1 (Figure 4). In clinical specimens, methylation at M1 was detected in 14% (3/22) of normal controls, 17% (4/23) of CIN1 lesions, 48% (13/27) of CIN3 lesions, and in all (20/20) SCCs (Figure 4). M2 methylation was found in 27% (6/22) of normal controls, 42% (11/26) of CIN1 lesions, 69% (20/29) of CIN3 lesions, and all (20/20) SCCs (Figure 4). Methylation at both regions was found in 5% (1/22) of normal controls, 9% (2/23) of CIN1 lesions, 38% (11/27) of CIN3 lesions, and 100% (20/20) of SCCs (Figure 4).

**Figure 5:**
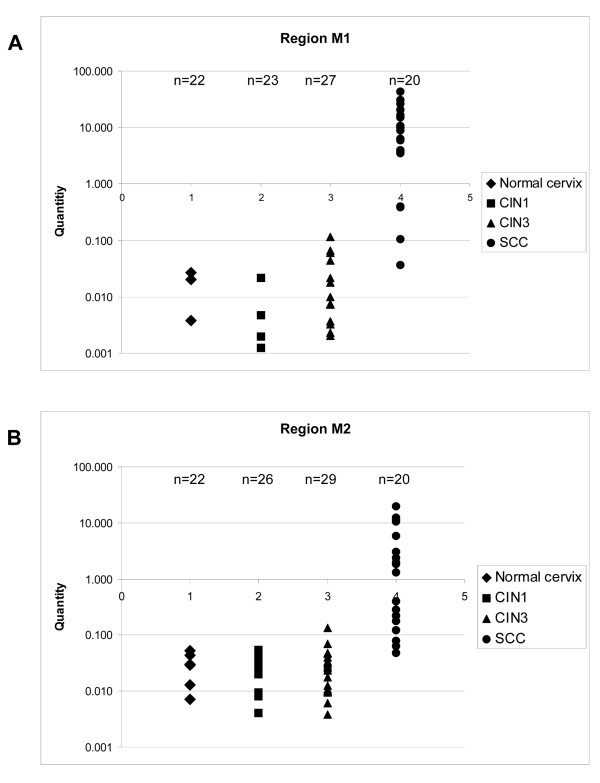
**Association between methylation and disease stage**. Scatter plots with on the y-axes levels of methylated DNA, on the x-axes the samples grouped for each disease stage (Note that only positive samples are given). (A) Region M1: -317 to -262 bp, relative to the ATG. (B) Region M2: +288 to +219 bp, relative to the ATG.

At both regions the level of methylation increased with the severity of cervical disease (Figure 5). Thus, not only the number of samples positive for methylation increased, but also the degree of methylation per sample increased with stage of disease.

## Discussion

Deregulated hTERT expression, resulting in the activation of telomerase, is a critical event during hrHPV-induced transformation, and is likely to mark a so-called point of no return during cervical carcinogenesis. To further elucidate the mechanisms underlying hTERT deregulation in HPV-transformed cells, both hTERT promoter activity and DNA methylation alterations were assessed in HPV-immortalized cells and cervical cancer cell lines as well as cervical (pre)cancerous lesions.

Using reporter constructs, we found that hTERT core promoter activity correlated to endogenous mRNA expression in most, though not all, cell lines. If hTERT expression is regulated mainly by transcription factor binding to the core promoter, reporter activity is expected to be correlated with endogenous hTERT expression. Our findings indicate, however, that sequences flanking the core promoter, particularly those spanning -499 to -297 and +88 to +348 (all relative to the ATG), inhibit basal hTERT promoter activity in HPV-immortalized cells and cervical carcinoma cell lines. Transcriptional repression by 5' sequences is in accordance with previous studies using cancer cell lines [6,7,37]. Transcriptional repressors with binding sites in this promoter distal region include WT1 (-358) [43], SP1 (-358, -323, +254) [44], EGR1 (-358) [45], and CTCF (+4) [18]. The latter two bind the hTERT promoter in cervical cancer cells [18,45]. Although transcriptional repression by sequences downstream of the core promoter confirms previous results, it cannot be completely ruled out that the reduced promoter activity of the hTERT-297/+357 construct is in part due to alternative splicing of the luciferase gene [37].

The partial disparity between endogenous hTERT expression and exogenous hTERT promoter activity prompted us to assess whether differences in epigenetic factors, particularly DNA methylation, may regulate hTERT expression in HPV-transformed cell lines, as was observed in other cell lines [11-18,39,42,46-48]. Most, though not all, studies found a positive correlation between hTERT methylation and hTERT expression.

By extensive bisulfite sequencing of HPV-transfected keratinocyte cell lines, a gradual increase in DNA methylation with progression from a mortal phenotype (represented by primary keratinocytes), via an immortal phenotype (represented by the HPV-immortalized cells) to a tumorigenic phenotype (represented by cervical cancer cells) was observed (Figure 3). Also in HPV-positive cervical samples representing (pre)malignant disease, an increase in methylation with progression of disease was detected at 2 of the 3 regions analyzed. Overall, methylation was less extensive in clinical specimens than in cell lines, which may be explained either by selection of cells containing methylated hTERT during culturing and/or the presence of normal cells in clinical specimens.

Remarkably, within region S3, containing exon 1, methylation was more abundant at putative binding sites for AP2, AP4 and MYOD [10]. As binding of AP2 can be inhibited by methylation [49], it is likely that binding of AP4 and MYOD is also methylation-sensitive. Although the beta isoform of AP2 activates hTERT transcription via the AP2 binding sites in the core promoter [50], other parts also contain AP2 binding sites, of which some may act repressive by means of other AP2 family-members (AP2α, γ, δ, ε).

Renaud *et al. *demonstrated that the methylation-sensitive repressor CTCF has two binding sites in the 5'end of hTERT, located at +4 to +39 and +422 to +440 (both relative to the ATG) [18]. Like in various human cancer cell lines and primary tumors, including HeLa cells and a cervical carcinoma [42], we also observed increased methylation of the first CTCF site in HPV-transformed cell lines and cervical SCC (Figure 3). However, the second CTCF site is less densily methylated than the surrounding sequences, implying that many alleles may still be able to recruit CTCF. Whether inhibition of CTCF binding to only the first site derepresses hTERT during HPV-mediated transformation remains to be determined. The high levels of methylation at both CTCF binding sites as detected in HeLa cells by Guilleret *et al. *[39], could not be confirmed in our studies, a feature that may be due to different HeLa subclones that were used [51].

Similar to CTCF, the transcriptional repressor Egr-1 binds to a CpG containing sequence located at -358 in the hTERT promoter (Figure 3). Interestingly, although Egr-1 acts as a transcriptional repressor of hTERT promoter-driven reporter constructs in cervical cancer cell lines, Egr-1 expression is elevated in hTERT positive cervical carcinomas [45]. Thus also the repressive activity of Egr-1 may be inhibited by DNA methylation in cervical cancer cells and contribute to hTERT expression, because in approximately half of the alleles in the HPV-immortalized cell lines and cervical cancer cell lines, the Egr-1 site was methylated (Figure 3A, CpG -42 - -46).

Recent studies have reported that although the hTERT promoter and coding sequence are frequently methylated in human tumors, a hypomethylated region around the transcription start site is believed to be sufficient to allow transcription [17,42]. Specific CpGs that were found to remain unmethylated in these studies are CpG -20 to CpG -12 [42] and CpG -19 to +14 [17]. Although we also observed only partial methylation around the transcription start site (at -58 bp from the ATG, between CpG -7 and -8), methylation was not decreased compared to the surrounding regions analyzed. In fact, in the SCCs we observed more methylation in region S2, spanning CpG -24 to +15, than in the two downstream regions. However, since the methylation patterns we observed are heterogeneous and dynamic, the unmethylated or partially unmethylated alleles may be expressed, as was suggested by Zinn *et al. *[17]. Support for this comes from the finding that active histone marks were particularly associated with unmethylated DNA and inactive histone marks generally, though less pronounced, with methylated DNA [17].

To assess the potential diagnostic value of hTERT methylation we performed qMSP analysis on a region in the hTERT promoter (-330 to -260 bp) and a second region in the first intron and second exon (+290 to +420 bp). With progression to cervical cancer a gradual increase in methylation was observed. Methylation at either the first or the second region was found in a relatively high percentage of normal controls (14% and 27%, respectively) and CIN1 lesions (17% and 37%, respectively). Together with the fact that hTERT mRNA expression is only rarely elevated in normal cervical cells and low grade cervical lesions [21,52], these findings may indicate that generally, methylation of both regions rather than either region is associated with deregulated hTERT expression.

Although to the best of our knowledge there are no reports on hTERT methylation in the complete spectrum of cervical squamous lesions, hTERT promoter methylation as detected by qMSP analysis using primers spanning -280 to -160 bp, relative to the ATG, has been detected in almost 60% of cervical carcinomas and none of normal cervical tissues [53]. The fact that we observed increased methylation of both regions analyzed in a marked subset of precursor lesions and in all cervical carcinomas indicates that hTERT methylation analysis may be of diagnostic value for the triage of hrHPV-positive women. Since past studies have shown that both telomerase activity and hTERT mRNA and protein levels in cervical scrapings only poorly reflect the status of these markers in the underlying lesions [54-57], hTERT methylation may be an attractive alternative marker.

## Conclusions

We demonstrated that CpG methylation of transcriptionally repressive sequences in the hTERT promoter and proximal exonic sequences is correlated to deregulated hTERT transcription in HPV-immortalized cells and cervical cancer cells. Analysis of cervical biopsies representing the full spectrum of cervical disease revealed that the detection of DNA methylation at these repressive regions may provide an attractive biomarker for early detection of cervical cancer.

## Competing interests

The authors declare that they have no competing interests.

## Authors' contributions

JW performed the cell culturing, luciferase assays and PCR reactions for bisulfite sequencing. JMK coordinated the bisulfite sequencing analysis and interpretation of sequencing results. JW designed the qMSP assays for hTERT. RMO prepared the clinical specimens for qMSP analysis and together with DC-K performed the qMSP assays. RDMS and PJFS designed the study and together with JMK participated in data analysis and interpretation. JW drafted the manuscript. RDMS, PJFS, CJLMM and JMK critically revised the manuscript. All authors have read and approved of the final manuscript.

## Pre-publication history

The pre-publication history for this paper can be accessed here:

http://www.biomedcentral.com/1471-2407/10/271/prepub
